# Patterns of Healthcare Expenditures among Older United States Adults with Pain and Different Perceived Health Status

**DOI:** 10.3390/healthcare9101327

**Published:** 2021-10-05

**Authors:** David Rhys Axon, Anisa Kamel

**Affiliations:** 1 Department of Pharmacy Practice and Science, College of Pharmacy, University of Arizona, Tucson, AZ 85721, USA; alkamel@pharmacy.arizona.edu; 2 Center for Health Outcomes and Pharmacoeconomic Research, College of Pharmacy, University of Arizona, Tucson, AZ 85721, USA

**Keywords:** pain, older adults, health status, health expenditures

## Abstract

The objective of this study was to assess the pattern of healthcare expenditures among United States (U.S.) adults aged ≥50 years with pain and annual total positive healthcare expenditures with different levels of perceived health. The study used the 2018 Medical Expenditure Panel Survey data. Unadjusted and adjusted linear regression models were used to compare logarithmically transformed total healthcare expenditures between those with excellent, very good, good, and fair/poor health. The a priori alpha value was 0.05. The study included 5123 U.S. adults aged ≥50 with self-reported pain (excellent = 8.9%, very good = 28.3%, good = 36.2%, fair/poor = 26.6%). In adjusted analyses, compared to fair/poor health, those with excellent health had the greatest adjusted reduction in expenditures (55% lower), followed by very good health (36.5% lower) and good health (24.9% lower). In conclusion, total positive healthcare expenditures were comparatively lower among those with better perceived health status for older (≥50 years) U.S. adults with pain that interfered with normal work in the past four weeks.

## 1. Introduction

Pain is one of the most common reasons United States (U.S.) adults seek medical care [1,2]. The prevalence of pain has increased in recent years and is associated with advanced age [3]. Pain is particularly prevalent among older adults aged ≥65 years [1], with half of older adults reporting bothersome pain in the past month in a 2011 study of 7601 U.S. adults aged ≥65 years [4]. Chronic pain affects 116 million Americans and costs the U.S. as much as $635 billion (in 2010 dollars) each year [5,6]. In 2017, an estimated $3.5 trillion was spent for healthcare expenditures due to chronic conditions in the U.S. [7]. This is concerning given the increasing population and the higher prevalence of chronic conditions among older adults [8]. It is estimated that by 2030, all baby boomers will be ≥65 years old, and the older adult population will outnumber children in the U.S. [9]. The increasing population of people with pain puts a significant economic burden on the healthcare system and healthcare providers; thus, it has been deemed a public health crisis [9]. 

In 2019, healthcare expenditure in the U.S. increased by nearly 5% [10]. Approximately 31% of this accounts for hospital costs, 15% for physician services, and 9.7% for prescription medications [10]. These rates have slightly increased in recent years [10]. With this continuously increasing healthcare expenditure, it is important to examine factors that may contribute to increased healthcare expenditure. 

Healthy People, a group of agencies and organizations that provides 10-year objectives for improving the nation’s health, has identified pain management as an area for improvement [11]. Their goal is “to reduce the proportion of patients suffering from untreated pain due to a lack of access to pain treatment, reduce the number of non-Food and Drug Administration (FDA)-approved pain medications, reduce serious injuries from the use of pain medicines, and reduce deaths from the use of pain medicines” [11]. Previous studies have already identified that individuals with pain report the use of multiple pain management strategies, which is burdensome to manage and associated with poor health status [12,13].

One important factor that may be associated with pain is the perceived health status of older adults. For example, a recent multi-country analysis found that, compared to good health, average-to-moderate self-rated health (odds ratio = 1.57) and poor health (odds ratio = 2.20) were significant predictors of pain among older adults [14]. 

However, there are currently limited data assessing how annual total positive healthcare expenditures of older U.S. adults with pain changes depending on perceived health status. It is important to investigate this topic given that these individuals are consumers of healthcare services in the U.S., the population of older U.S. adults with pain is increasing, and there is an ever-increasing demand for healthcare resources. Knowledge of how health status is associated with annual total positive healthcare expenditures may help indicate the need for policies or interventions to maintain or improve the health of older U.S. adults with pain. In order to conduct this investigation, this study used the Medical Expenditure Panel Survey (MEPS) dataset to examine how annual total positive healthcare expenditures of older U.S. adults with pain changes depending on perceived health status. 

## 2. Materials and Methods

This study utilized a cross-sectional design using the 2018 MEPS data. MEPS uses the sampling frame from the prior year’s National Health Interview Survey (NHIS), and the sample can be weighted during analyses to provide nationally representative estimates of the non-institutionalized U.S. population [15]. There are three main components to MEPS: MEPS household component (MEPS-HC), MEPS medical provider component (MEPS-MPC), and MEPS insurance component (MEPS-IC) [15]. Household data are obtained by surveying primary sampling units (i.e., households) over a two-year period through five interview rounds (panels) by workers on behalf of the Agency for Healthcare Research and Quality [15]. MEPS-HC data include demographic characteristics, health conditions, health status, use of medical services, access to care, income, and healthcare expenditure data, among others. Data from the MEPS-MPC and MEPS-IC are used to supplement the MEPS-HC data to improve the reliability and validity of the dataset [15]. This study used the most up-to-date data available at the time of the study, which was obtained from the 2018 full-year consolidate data file (MEPS-HC-209). The 2018 file contains data obtained from panel 22 (interview rounds 3, 4, 5) and panel 3 (interview rounds 1, 2, 3) [16]. All participants provided verbal informed consent.

Subjects from the 2018 MEPS dataset were included if they: were alive during the 2018 calendar year; were aged ≥50 years; reported having pain that interfered with normal work in the past four weeks; and had positive annual total healthcare expenditures.

The independent variable was perceived health status. Categories included were excellent, very good, good, and fair/poor health [16]. The control variables included age (50–64 or ≥65 years), gender (male or female), race (white or other), census region (Northeast, Midwest, South, or West), marital status (married or other), education (less than high school, high school, or more than high school), employment (employed or unemployed), income (poor/near poor/low or middle/high), health insurance (private, public, or uninsured), frequent exercise (yes or no), current smoker (yes or no), functional limitations (yes or no), mental health status (excellent/very good/good or fair/poor), pain severity (quite a bit/extreme or little/moderate), and chronic conditions (<3, 3–5, >5) [16]. The dependent variable was annual total positive healthcare expenditures [16].

Descriptive statistics were computed to compare the baseline characteristics of the groups using chi-square tests. Unadjusted and adjusted linear regression models were constructed using logarithmically transformed data (due to the non-linear nature of healthcare expenditure data) to assess differences in annual total positive healthcare expenditures between groups (excellent health, very good health, good health, fair/poor health), with fair/poor health serving as the reference group. The assumptions of linear regression (homoscedasticity, independent observations, linearity, normality, no multicollinearity) were assessed and found to be acceptable. The unadjusted model contained only the independent variable (health status), whereas the adjusted model included the independent variable and all the control variables described above. The percent difference between healthcare expenditure groups was calculated using semi-logarithmic equations, again using fair/poor health as the reference group. Analyses accounted for the complex survey design of MEPS. Nationally representative estimates were obtained using the relevant weighting variable, and variance estimates were calculated using the Taylor-series linearization method. The alpha level (selected a priori) to determine statistical significance was 0.05. Analyses were conducted using PROC SURVEY commands in SAS Studio (SAS Institute Inc., Cary, NC, USA).

## 3. Results

Of the 30,461 subjects available in the 2018 MEPS dataset, 5123 met eligibility criteria and were included in the study. The prevalence of excellent health was 8.9% (95% confidence interval (CI) = 7.7, 10.0), very good health was 28.3% (95% CI = 26.6, 30.0), good health was 36.2% (95% CI = 34.4, 38.0), and fair/poor health was 26.6% (95% CI = 24.9, 28.3).

Most study subjects were ≥65 years of age, female, white, married, had more than high school education, unemployed, middle/high income, had private health insurance, did not frequently exercise, were not current smokers, had no functional limitation, had excellent/very good/good mental health status, had little/moderate pain severity, and had 3–5 chronic health conditions. Participants most commonly resided in the southern census region. There were statistically significant differences (*p* < 0.05) between all characteristics except age (*p* = 0.6338) and gender (*p* = 0.7751). See Table 1.

Models adjusted for the following variables: age, gender, race, census region, marital status, education, employment, income, health insurance, frequent exercise, current smoker, functional limitation, mental health status, pain severity, and chronic conditions.

**Figure 1:**
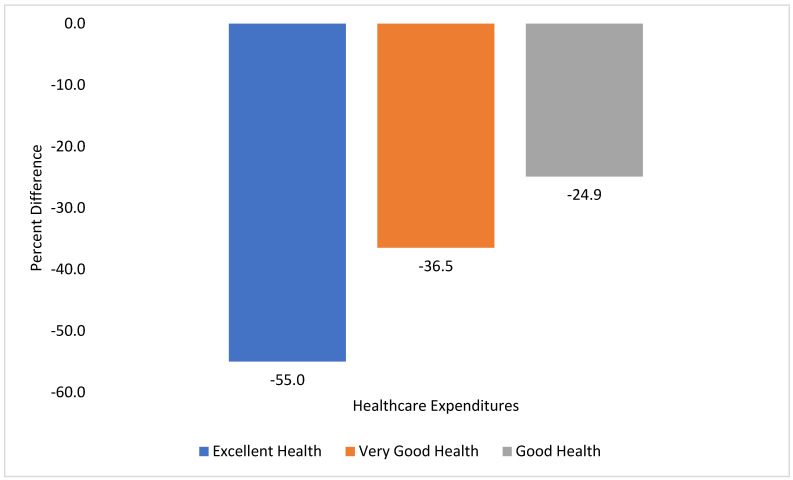
Percent difference in 2018 adjusted annual total positive healthcare expenditures for United States older adults (age ≥50 years) with self-reported pain in the past four weeks. Percent change represents the difference in adjusted 2018 healthcare expenditures between United States older adults with self-reported pain with fair/poor health (reference group) and excellent health (blue bars), very good health (orange bars), and good health (grey bars).

In both unadjusted and adjusted analyses, excellent health had the greatest reduction in annual total positive healthcare expenditures versus fair/poor health (55% lower), followed by very good health versus fair/poor health (36.5% lower), and then good health versus fair/poor health (24.9% lower). See Table 2 and Figure 1.

## 4. Discussion

The key finding from this study was that, in adjusted linear regression analyses, older U.S. adults with pain and better perceived health status had lower annual total positive healthcare expenditures than those with relatively poorer perceived health. Compared to those with fair/poor perceived health status, there was a 55%, 36.5%, and 24.9% decrease in total expenditures for excellent, very good, and good perceived health status, respectively. There are limited up-to-date data of annual total positive healthcare expenditures for older U.S. adults with pain published in the literature. Therefore, the findings from the current study add contemporary new knowledge about the association between perceived health status and annual total positive healthcare expenditures in this population. According to a study that used 2016 MEPS data, U.S. adults aged 55 and older accounted for over half of the total healthcare spending, and among those who reported fair/poor health status, 10% of the group accounted for over half the total expenditure [17]. This emphasizes the importance of understanding the nature of healthcare expenditures among older U.S. adults and the need improve poor health and maintain good health in order to control costs. Another study identified that health status variables are a strong predictor of healthcare expenditures [18]. The current study also found that among those with poor perceived health status, factors including increased chronic conditions, increased pain severity, functional limitations, and decreased exercise frequency were more prevalent compared to those with excellent perceived health status. Similar findings were observed among U.S. adults aged 65 years or younger using data from the 1997–2003 Behavioral Risk Factor Surveillance System (BRFSS), which suggested that the proportion of U.S. adults aged 65 years or younger with excellent/very good perceived health status declined from 64% to 56% [19]. During the same six-year study period, healthcare expenditure nearly doubled per capita, increasing from $3468 to $7421 [19]. The findings of the current study may be partially influenced by pain severity. For instance, one study using 2008–2011 MEPS data found that pain (which may be a contributing factor for perceived health status) was associated with healthcare expenditures; moderate pain was associated with a $3707 increase, and severe pain was associated with a $5804 increase in healthcare expenditure as compared to no pain [20]. The current study showed older adults with greater pain severity were more likely to have poor/fair perceived health status. Of those with excellent perceived health status, only 8.3% reported quite a bit/extreme pain, while 51.4% reported quite a bit/extreme pain in the fair/poor health status population. 

The findings of the current study combined with the limited existing knowledge highlight the need for greater action to prevent older U.S. adults with pain from a deteriorating health status to help prevent an associated increase in annual total positive healthcare expenditures. Likewise, there is a need for health promotion interventions to improve the health of those who already perceive their health as fair or poor in order to help reduce associated annual total positive healthcare expenditures. This will become ever more relevant as the proportion of older adults with health conditions in the population continues to increase, and healthcare resources (including available money) become increasingly strained.

Limitations are present in this study. MEPS involved self-reported data that may lead to bias and is reliant on participants accurately reporting their data. Several factors included in the study that may contribute to perceived health status are subjective factors that cannot be objectively measured, such as mental health status and pain severity. The study only included individuals with annual total positive healthcare expenditures; thus, those older adults with pain who had no healthcare expenditures were not captured in this study. In addition, due to the MEPS sampling framework, the results of this study are generalizable only to the non-institutionalized, civilian population.

## 5. Conclusions

In adjusted analyses, the findings from this study demonstrated excellent health had the greatest reduction in annual total positive healthcare expenditures compared to those with fair/poor health, followed by very good perceived health and then good perceived health status among the older U.S. adult population with self-reported pain. These findings add new knowledge to help researchers better understand the relationships between perceived health status and healthcare expenditures among older adults with pain.

## Figures and Tables

**Table 1:** Sample characteristics of United States older adults (age ≥50 years) with self-reported pain in the past four weeks, stratified by self-reported health status.

Characteristics	Excellent Health (*N =* 417) % (95% CI)	Very Good Health (*N* = 1320) % (95% CI)	Good Health (*N* = 1875) % (95% CI)	Fair/Poor Health (*N* = 1511) % (95% CI)	*p*
Age (years)					0.6338
50–64	48.7 (42.0, 55.3)	46.9 (43.4, 50.5)	49.9 (46.9, 52.8)	48.0 (44.7, 51.2)	
≥65	51.3 (44.7, 58.0)	53.1 (49.5, 56.6)	50.1 (47.2, 53.1)	52.0 (48.8, 55.3)	
Gender					0.7751
Male	46.3 (41.4, 51.2)	43.6 (41.0, 46.3)	44.8 (42.4, 47.1)	43.8 (40.8, 46.8)	
Female	53.7 (48.8, 58.6)	56.4 (53.7, 59.0)	55.2 (52.9, 57.6)	56.2 (53.2, 59.2)	
Race					<0.0001
White	85.5 (81.9, 89.0)	84.7 (82.6, 86.8)	78.8 (76.1, 81.5)	78.3 (75.5, 81.1)	
Other	14.5 (11.0, 18.1)	15.3 (13.2, 17.4)	21.2 (18.5, 23.9)	21.7 (18.9, 24.5)	
Census region					0.0407
Northeast	13.3 (8.8, 17.8)	18.9 (15.7, 22.1)	17.6 (14.7, 20.4)	16.3 (13.2, 19.4)	
Midwest	22.1 (14.9, 29.3)	22.8 (19.5, 26.1)	22.4 (19.7, 25.1)	20.5 (17.4, 23.7)	
South	37.4 (30.8, 43.9)	34.6 (30.7, 38.5)	38.0 (34.9, 41.2)	43.4 (39.7, 47.1)	
West	27.2 (22.6, 31.9)	23.7 (20.7, 26.8)	22.0 (19.0, 25.1)	19.8 (17.0, 22.6)	
Marital status					<0.0001
Married	63.7 (57.9, 69.5)	63.8 (60.4, 67. 3)	54.7 (51.8, 57.7)	49.4 (45.9, 53.0)	
Other	36.3 (30.5, 42.1)	36.2 (32.7, 39.6)	45.3 (42.3, 48.2)	50.6 (47.0, 54.1)	
Education					<0.0001
Less than high school	9.1 (6.1, 12.1)	7.2 (5.7, 8.7)	13.2 (11.5, 14.9)	23.5 (20.3, 26.7)	
High school	23.3 (18.6, 28.0)	26.7 (23.9, 29.5)	33.7 (31.2, 36.3)	34.8 (31.7, 37.9)	
More than high school	67.6 (61.9, 73.2)	66.1 (62.9, 69.3)	53.0 (50.3, 55.8)	41.7 (37.6, 45.8)	
Employment					<0.0001
Employed	53.9 (47.7, 60.2)	50.3 (46.7, 53.9)	41.2 (38.3, 44.1)	22.1 (18.9, 25.3)	
Unemployed	46.1 (39.8, 52.3)	49.7 (46.1, 53.3)	58.8 (55.9, 61.7)	77.9 (74.7, 81.1)	
Income					<0.0001
Poor/near poor/low	16.9 (13.1, 20.6)	18.5 (16.0, 21.0)	31.8 (29.3, 34.3)	47.9 (43.9, 51.8)	
Middle/high	83.1 (79.4, 86.9)	81.5 (79.0, 84.0)	68.2 (65.7, 70.7)	52.2 (48.2, 56.1)	
Health insurance					<0.0001
Private	66.8 (60.8, 72.9)	69.8 (66.4, 73.1)	57.0 (54.2, 59.9)	42.2 (39.0, 45.4)	
Public	30.4 (24.5, 36.2)	27.7 (24.6, 30.8)	39.7 (36.9, 42.6)	54.4 (51.3, 57.5)	
Uninsured	2.8 (1.2, 4.3)	2.5 (1.5, 3.5)	3.2 (2.3, 4.2)	3.4 (1.9, 4.9)	
Frequent exercise					<0.0001
Yes	64.1 (58.2, 70.0)	51.6 (47.9, 55.3)	39.9 (37.1, 42.7)	27.3 (24.4, 30.2)	
No	35.9 (30.0, 41.8)	48.4 (44.7, 52.1)	60.1 (57.3, 62.9)	72.7 (69.8, 75.6)	
Current smoker					<0.0001
Yes	6.8 (3.8, 9.8)	10.7 (8.8, 12. 6)	18.0 (16.0, 20.1)	22.0 (19.0, 25.0)	
No	93.2 (90.2, 96.2)	89.3 (87.4, 91.2)	82.0 (79.9, 84.0)	78.0 (75.0, 81.0)	
Functional limitations					<0.0001
Yes	12.5 (8.7, 16.4)	23.9 (20.9, 26.8)	39.6 (37.0, 42.2)	64.5 (61.7, 67.3)	
No	87.5 (83.6, 91.3)	76.1 (73.2, 79.1)	60.4 (57.8, 63.0)	35.5 (32.7, 38.3)	
Mental health status					<0.0001
Excellent/very good/good	97.7 (96.2, 99.1)	96.6 (95.4, 97.9)	92.8 (91.5, 94.1)	60.1 (57.3, 62.9)	
Fair/poor	2.3 (0.9, 3.8)	3.4 (2.1, 4.6)	7.2 (5.9, 8.5)	39.9 (37.1, 42.7)	
Pain severity					<0.0001
Quite a bit/extreme	8.3 (5.6, 11.1)	9.9 (8.2, 11.7)	22.4 (20.2, 24.5)	51.4 (48.3, 54.5)	
Little/moderate	91.7 (99.9, 94.4)	91.0 (88.3, 91.8)	77.6 (75.5, 79.8)	48.6 (45.5, 51.7)	
Chronic conditions					<0.0001
<3	3.7 (2.0, 5.4)	7.9 (6.1, 9.6)	12.4 (10.4, 14.3)	24.8 (21.8, 37.7)	
3–5	31.0 (25.6, 36.4)	43.3 (40.3, 46.2)	50.2 (47.7, 52.7)	52.7 (49.6, 55.8)	
>5	65.3 (59.7, 70.9)	48.9 (45.8, 51.9)	37.4 (35.0, 39.8)	22.5 (19.9, 25.1)	

Abbreviations: %, percentage; CI, confidence interval. Chi-square tests were used to determine differences between groups.

**Table 2:** Parameter estimates and percent changes from unadjusted and adjusted linear regression models for self-reported health status among United States older adults (age ≥50 years) with self-reported pain in the past four weeks, using logged annual total positive healthcare expenditures.

Perceived Health Status	Unadjusted			Adjusted		
	Beta Estimate (Standard Error)	*p*	Percent Change	Beta Estimate (Standard Error)	*p*	Percent Change
Excellent health	−1.29 (0.10)	<0.0001	−72.4	−0.80 (0.11)	<0.0001	−55.0
Very good health	−0.76 (0.07)	<0.0001	−53.4	−0.45 (0.07)	<0.0001	−36.5
Good health	−0.53 (0.06)	<0.0001	−40.9	−0.29 (0.06)	<0.0001	−24.9
Fair/poor health	Reference			Reference		

## Data Availability

The data presented in this study are available on request from the corresponding author.
